# Fiber-modified adenovirus-mediated suicide gene therapy can efficiently eliminate bladder cancer cells *in vitro* and *in vivo*

**DOI:** 10.18632/oncotarget.12324

**Published:** 2016-09-28

**Authors:** De-Gui Wang, Mei-Jun Zhao, Yong-Qiang Liu, Xiang-Wen Liu, Hai-Tao Niu, Yan-Feng Song, Ying-Xia Tian

**Affiliations:** ^1^ Department of Anatomy and Histology, School of Basic Medical Sciences, Lanzhou University, Lanzhou 730000, China; ^2^ Department of Internal Medicine, Gansu Provincial Academic Institute for Medical Research, Lanzhou 730050, China; ^3^ Department of Urology, Affiliated Hospital of Qingdao University, Qingdao 266071, China

**Keywords:** gene therapy, bladder cancer, suicide gene, adenovirus

## Abstract

Adenovirus-mediated gene therapy is a promising strategy for bladder cancer treatment. However, the loss of the coxsackie and adenovirus receptor (CAR) in bladder cancer cells decreases the infection efficiency of the therapeutic adenovirus. In this study, we constructed an Arg-Gly-Asp (RGD)-modified adenovirus, RGDAd-UPII-TK, that carries a suicide gene called HSV-TK that is driven by a human UPII promoter. Then, we tested the bladder cancer specificity of the UPII promotor and the expression of the HSV-TK protein. Additionally, we observed a potent cytotoxic effects of RGDAd-UPII-TK and ganciclovir (GCV) on bladder cancer as demonstrated by reduced cell survival and morphology changes *in vitro*. Furthermore, we confirmed that RGDAd-UPII-TK in combination with a GCV injection could significantly reduce the established T24 tumor growth and increase apoptosis *in vivo*. Altogether, our results indicated that the recombinant adenovirus RGDAd-UPII-TK could target bladder cancer through valid gene therapy.

## INTRODUCTION

Bladder cancer is the second-most common cause of genitourinary cancer-related mortality in humans worldwide [1], whereas the current therapeutic strategies, including surgery excision, chemotherapy, radiotherapy and immunotherapy, have multiple severe side effects and unfavorable prognoses [2–4]. Currently, precision medicine for personalized cancer treatment has been well-developed with a series of positive feedback from numerous patients [5]. In particular, diverse adenoviral vectors for gene therapy represent a promising strategy based on laboratory experiments as well as clinical trials [6, 7]. The recombinant adenovirus is an efficient gene delivery system and can be easily produced with high titers [8]. Among the more than 50 serotypes detected in humans, adenovirus types 2 and 5, which belong to the subgroup C family, are commonly used for oncolytic therapy because the viruses replicate episomally without host genome insertion [9]. In our study, we utilized a special replication-deficient adenovirus type 5 (Ad5) that can minimize the danger of host genome instability and tumorigenesis. Moreover, Ad5 can transfer genes into both dividing and non-dividing cells [10]. Meanwhile, the bladder is an anatomically accessible organ for direct intravesical administration of adenoviral vectors and therapeutic agents rather than systemic administration, so clearance by the immune system can be avoided [11].

However, there are still some obstacles regarding adenovirus-mediated gene therapy. Previous studies have demonstrated that the coxsackie and adenovirus receptor (CAR), which plays an important role in virus internalization through recognizing and binding the viral fiber knob [12], is downregulated or even abrogated in several human bladder cancer cell lines [13]. Additionally, the selectivity of the therapeutic adenovirus for bladder cancer is deficient. To overcome these shortcomings, we generated a novel fiber-modified recombinant adenovirus, RGDAd-UPII-TK, that contains a Arg-Gly-Asp (RGD) motif in the HI loop of the fiber and carries a suicide gene encoding for the herpes simplex virus-thymidine kinase (HSV-TK) driven by the bladder-specific human Uroplakin II (UPII) promoter inserted upstream. RGD is the cell attachment site for several surrounding extracellular matrix (ECM) proteins, including fibronectin, which can interact with cells by binding to integrins, which are transmembrane cell surface receptors [14, 15]. Therefore, we assumed that using RGD to modify the adenoviral fiber may prompt the CAR-negative bladder cancer cells to internalize these gene-delivery vectors.

In our previous studies, we constructed a recombinant oncolytic adenovirus Ad-UPII-E1A that targeted bladder cancer cells for E1A expression under the control of the UPII promotor [16, 17]. The oncolytic effects of Ad-UPII-E1A are based on adenoviral replication, which is also limited. To investigate the effects, we explored whether it was feasible to enhance adenoviral cytotoxicity by suicide gene therapy. Our studies evaluated the selectivity and cytotoxicity of the recombinant adenovirus RGDAd-UPII-TK for bladder cancer both *in vitro* and *in vivo*. HSV-TK can convert ganciclovir (GCV) into GCV-monophosphate (GCV-MP), which will be recognized by cellular kinases and then converted into GCV-triphosphate (GCV-TP), which is a guanine nucleoside analogue that may cause DNA chain termination and subsequent apoptosis cell death. Additionally, GCV-MP can passively diffuse into neighboring cells to exert a “bystander effect”, which can expand the elimination of tumor cells [18–21].

## RESULTS

### Construction of the fiber-modified adenovirus RGDAd-UPII-TK

**Figure 1:**
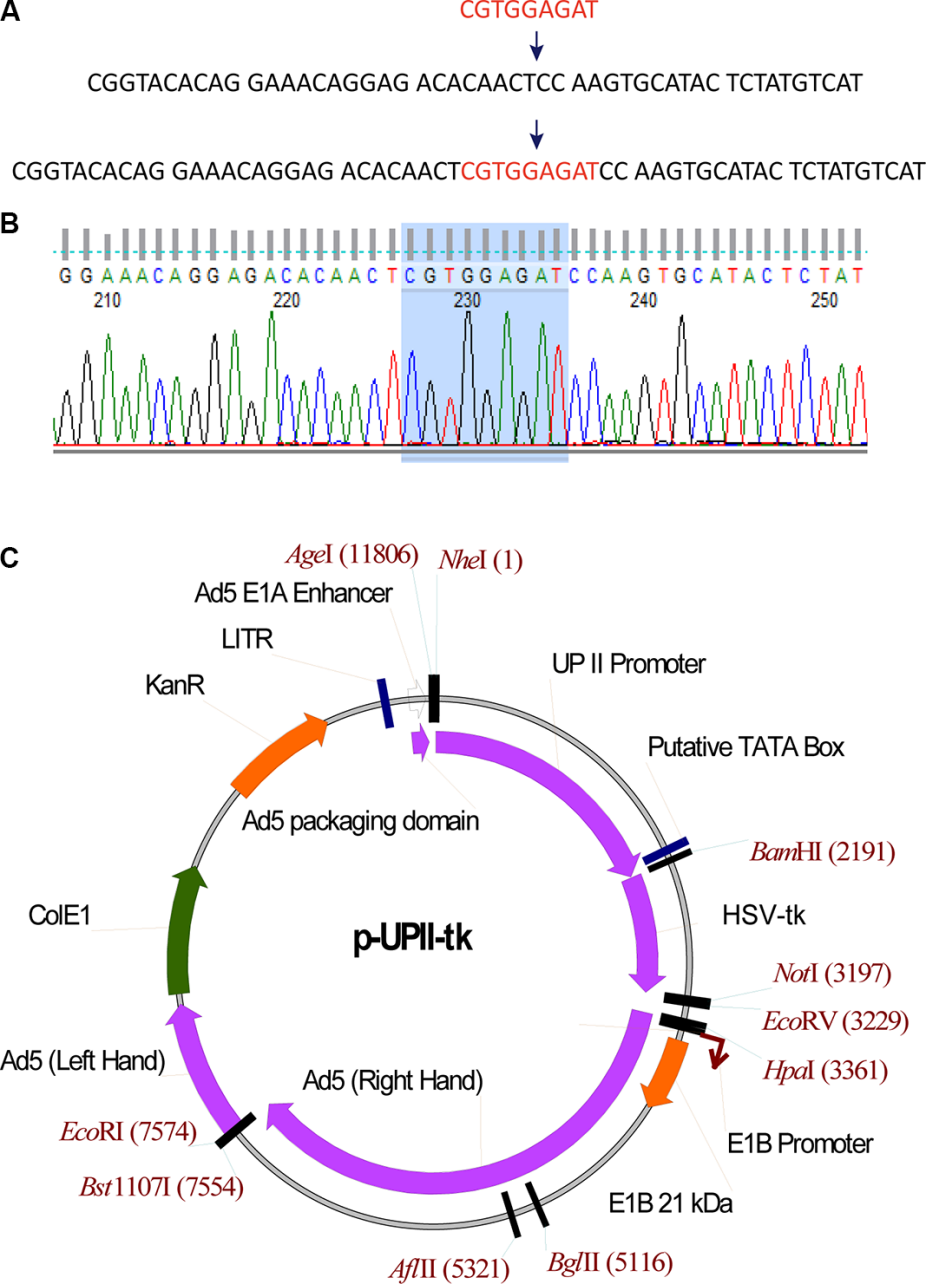
Modification and Identification of RGDAd-UPII-TK (**A**) Schematic insertion of the RGD sequence into an adenoviral plasmid at the indicated site. (**B**) Sequencing results of the constructed plasmid. The shadow region shows the correct insertion of RGD sequence into pAdEasy-1. (**C**) Schematic construction of adenovirus shuttle plasmid. The plasmid profile shows the location of the UPII promoter and the HSV-TK gene.

To increase the tropism of the virus against CAR-deficient bladder cancer cells, we modified the HI loop of the adenoviral fiber. Overlapping PCR was performed to clone the RGD sequence into the HI loop coding region of pAdEasy-1, which is an adenoviral backbone plasmid (Figure 1A). The modified plasmid was called pAd-RGD. The sequencing results are shown in Figure 1B.

To achieve bladder cancer-specific suicide gene therapy, we inserted the UPII promotor and HSV-TK gene into a shuttle plasmid to generate the plasmid p-UPII-TK (Figure 1C). We also constructed p-UPII-LUC as a reporter vector and p-UPII-Null as a control. The recombinant adenovirus vectors Ad-UPII-LUC, RGDAd-UPII-LUC, RGDAd-UPII-TK and RGDAd-UPII-Null were established through homologous recombination, and the adenoviruses were amplified and packaged in HEK 293 cells.

### UPII promoter drove HSV-TK gene expression in bladder cancer cells

**Figure 2:**
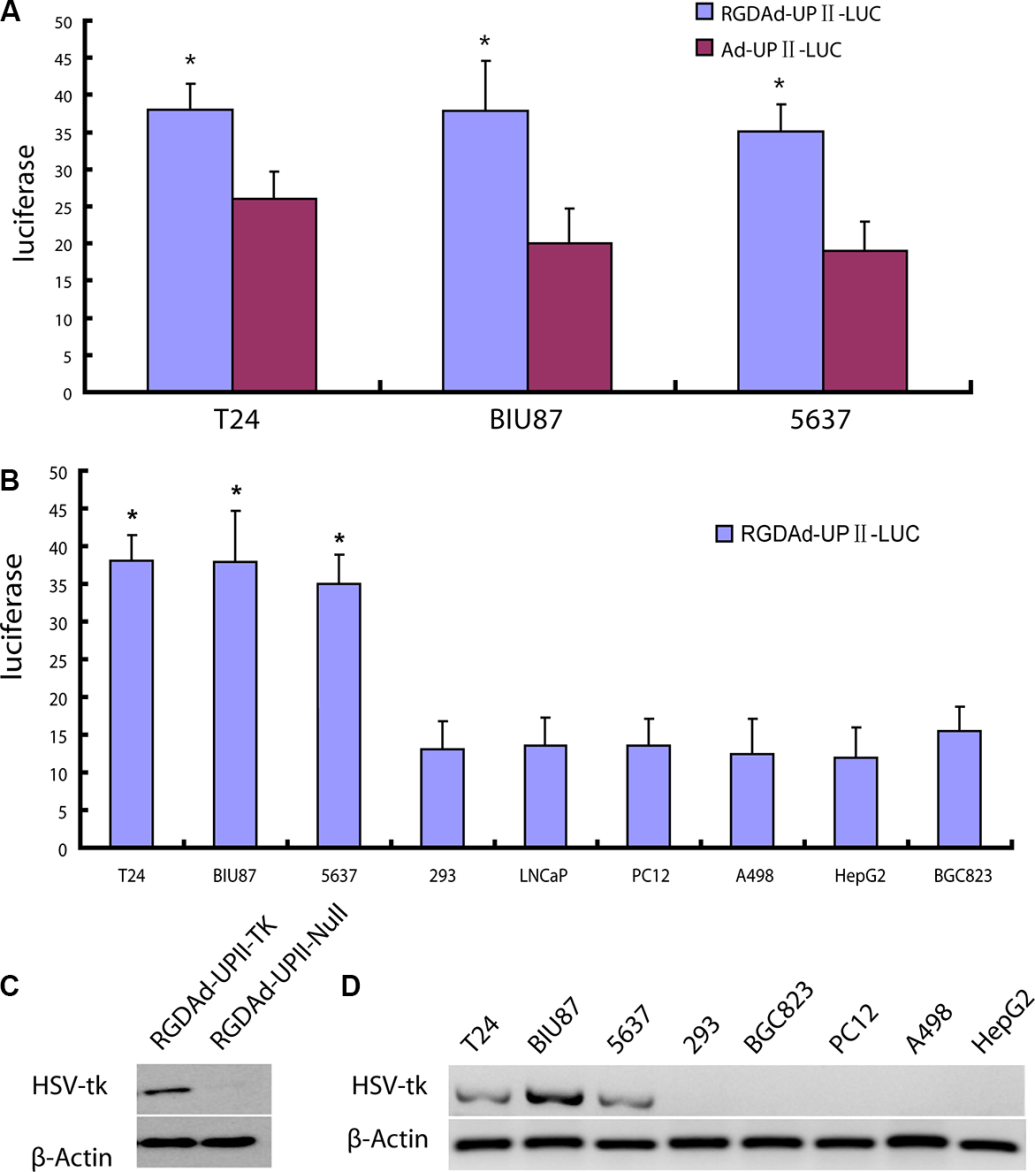
Analysis of HSV-TK gene expression with modified Adenovirus (**A**) Luciferase expression levels of T24 that were infected with RGDAd-UPII-Luc and Ad-UPII-Luc. Luc activities were measured after the infection for 24 hours. The data are presented as the mean±SD of at least 3 independent experiments. * indicates statistical significance (*P < 0.05). (**B**) Luciferase expression levels of the indicated cell lines infected with RGDAd-UPII-Luc. (**C**) HSV-TK expression in adenovirus infected bladder cancer cells. T24 cells transfected with RGDAd-UPII-TK or RGDAd-UPII-Null were analyzed by Western blot for HSV-TK. β-actin was used as a loading control. (**D**) Western blot analysis of HSV-TK protein expression in bladder cancer cell lines and non-bladder cancer cell lines. The cells were collected and lysed 24 hours after the infection.

We have constructed RGDAd-UPII-TK, which carries the suicide gene downstream of the UPII promotor. First of all, we compared the infection activity of modified RGD peptide through a luciferase reporter assay in the three bladder cancer cell lines, including T24, BIU-87, and 5637. The data exhibited that the luciferase activity of RGDAd-UPII-Luc infected cells were all significantly higher compared to the Ad-UPII-Luc infected cells, which suggested that the RGD-modified adenovirus can increase infectivity for bladder cancer cells (Figure 2A). To further assess the bladder cancer selectivity of the virus, a luciferase reporter assay was performed after T24, BIU-87, 5637, and other non-bladder cells, including Human Embryonic Kidney 293, LNCaP (prostate cancer), PC12 (pheochromocytoma), A498 (renal cancer), HepG2 (liver cancer), and BGC823 (gastric cancer), were infected with RGDAd-UPII-Luc and then incubated for 24 h. The results showed a higher luciferase activity in bladder cancer cell lines compared to non-bladder cancer cells (Figure 2B).

To determine the infectivity of the recombinant adenovirus RGDAd-UPII-TK and the efficacy of HSV-TK protein expression, Western blotting was performed 24 h after T24 cells were infected with RGDAd-UPII-TK. The results showed that HSV-TK expression was markedly positive in the cells infected with RGDAd-UPII-TK but was absent in RGDAd-UPII-Null infected control cells. These results suggested that the recombinant adenovirus-mediated gene delivery was valid, and the UPII promotor could considerably drive HSV-TK gene expression in bladder cancer cells (Figure 2C). Then we utilized the previous 8 cell lines to examine the bladder cancer specificity of the recombinant RGDAd-UPII-TK vector at the protein level. Western blotting showed that HSV-TK expression was significantly higher in the bladder cancer cell lines compared to other non-bladder cancer cell lines (Figure 2D).

### RGDAd-UPII-TK eliminated bladder cancer cells *in vitro* when combined with GCV

**Figure 3:**
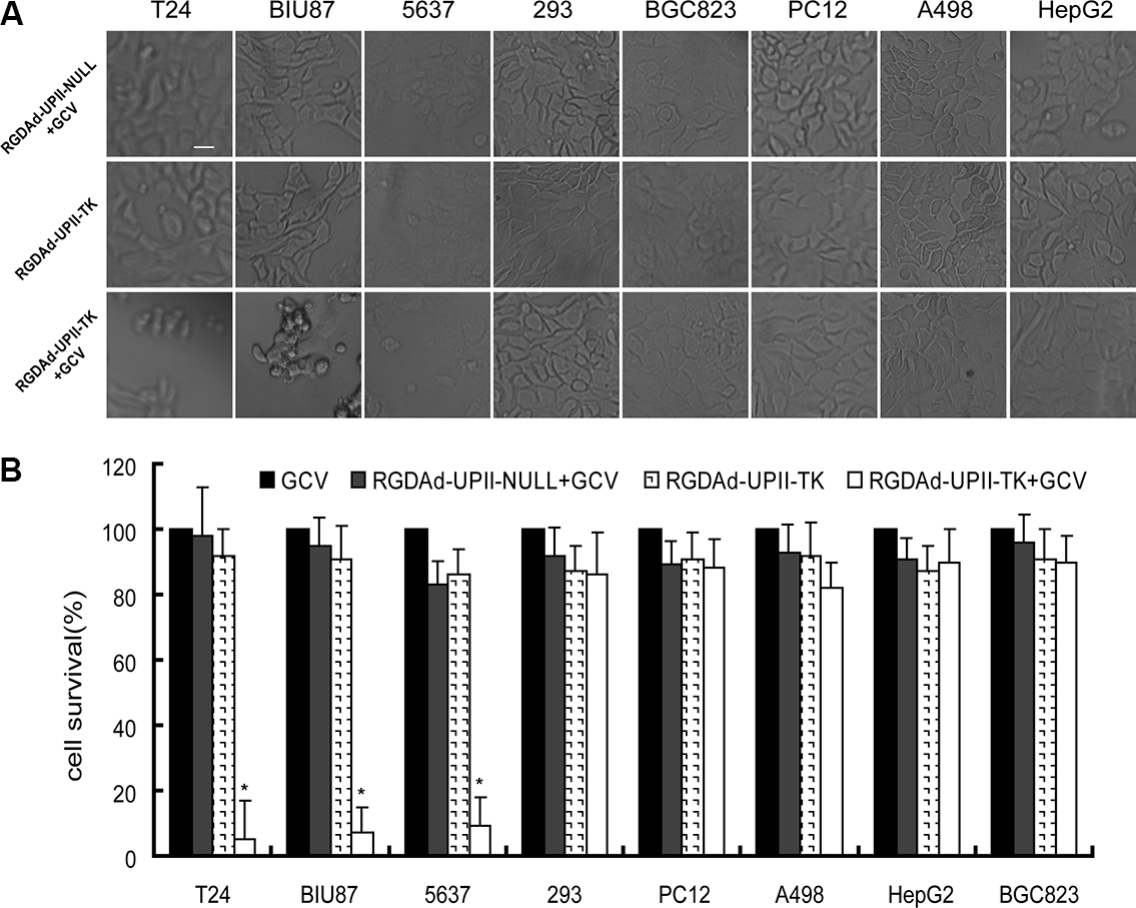
RGDAd-UPII-TK in combination with GCV treatment can eliminate bladder cancer cells efficiently *in vitro* (**A**) Cell quantities and morphology changes after infection with RGDAd-UPII-TK or RGDAd-UPII-Null in combination with GCV treatment. Scale bar, 10 μm. (**B**) Cell viabilities were examined by MTT assay with three replicates for each condition. The relative cell viability is shown as a ratio relative to the GCV-treated control, and all data are presented as the mean + S.D. * indicates statistical significance (*P < 0.05).

Here, we demonstrated that the suicide gene HSV-TK could be highly expressed in bladder cancer cells under the control of the UPII promotor. Then, we examined the cytotoxic effect of RGDAd-UPII-TK combined with GCV treatment. Bladder cancer cells and non-bladder cancer cells were cultured and infected with RGDAd-UPII-TK or RGDAd-UPII-Null. Subsequently, the infected cells were treated with or without GCV for 12 h. We observed that in the RGDAd-UPII-TK combined with GCV-treated group, multiple bladder cancer cells were eliminated, and the remaining cells became round and appeared to have more vacuoles in the cytoplasm compared to the other non-bladder cancer cells. However, in the RGDAd-UPII-Null in combination with GCV treated and RGDAd-UPII-TK alone control groups, the cells grew and proliferated well with a normal morphology (Figure 3A).

To further investigate the cytotoxic effects and tissue specificity of this anticancer strategy, we employed the 8 cancer cell lines in an MTT assay. The data showed that when infected with the recombinant adenovirus RGDAd-UPII-TK in combination with GCV treatment, T24, BIU87 and 5637 cells showed a dramatic decrease in cell viability, whereas the other non-bladder cancer cells showed no obvious cell death. However, when treated with GCV or RGDAd-UPII-TK alone, or infected with RGDAd-UPII-Null combined with GCV treatment, the cell lines displayed high viability (Figure 3B). The above experiments confirmed that RGDAd-UPII-TK and GCV treatment could eliminate bladder cancer cells efficiently *in vitro*.

### RGDAd-UPII-TK suppressed bladder cancer growth *in vivo*

**Figure 4:**
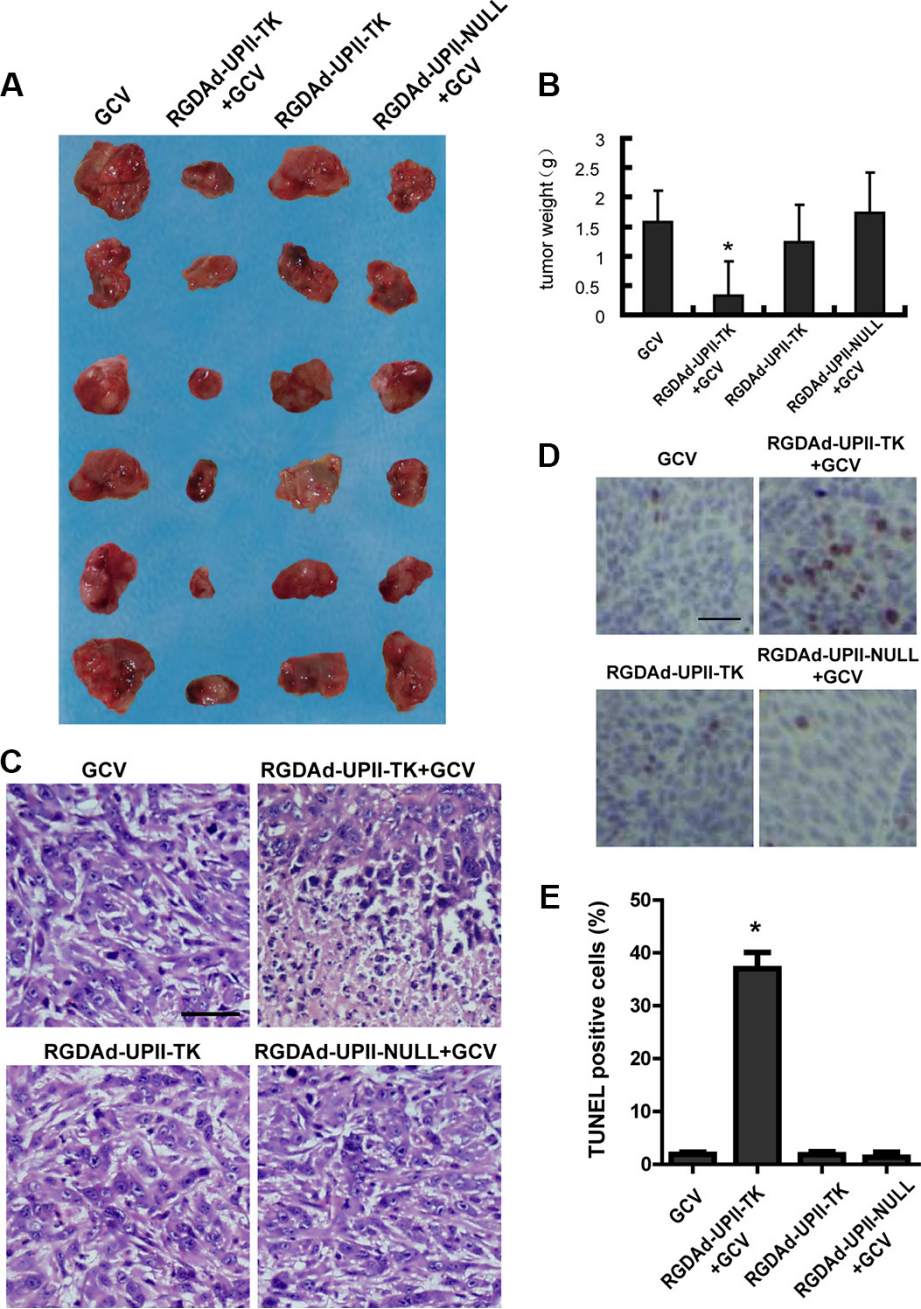
The effect of RGDAd-UPII-TK infection on T24 implanted tumors in nude mice (**A**) Photograph of subcutaneously implanted tumors excised from the nude mice. The tumor sizes in each group were compared. (**B**) The tumor weights in each group were measured and compared. (**C**) H&E staining of the implanted tumors after the indicated treatment. Scale bar, 50 μm. (**D**) Images of TUNEL staining of the excised tumor tissues showing the apoptotic cells. The cells were counterstained with hematoxylin. Scale bar, 50 μm. E. Statistical results of TUNEL-positive cells per field. All data are presented as the mean + S.D. * indicates statistical significance (*P < 0.05).

For *in vivo* anti-cancer experiments, we chose the T24 bladder cancer cell line to establish tumor-bearing nude mice models. When palpable tumors were established, the mice were randomly divided into four groups (six mice per group). Adenoviruses RGDAd-UPII-TK or RGDAd-UPII-Null were directly injected into the tumors every two days followed by intraperitoneal injection of GCV. After six days of treatment, the mice were sacrificed, and the tumors were excised to measure their sizes and weights. The results revealed that the tumor growth of the RGDAd-UPII-TK in combination with GCV treated group was significantly inhibited compared to the other three mock-treated control groups (Figure 4A, 4B).

Then we examined the pathological change of the excised tumor tissues using hematoxylin-eosin (H&E) staining. We observed that the bladder cancer cells in the RGDAd-UPII-TK and GCV-treated groups suffered extensive cell death compared to the control groups (Figure 4C). Additionally, a TUNEL assay was performed to detect apoptosis. The results showed that the RGDAd-UPII-TK and GCV-treated tumor tissues presented a significantly higher apoptotic rate than other three control groups (Figure 4D, 4E). Altogether, the results suggested that the recombinant adenovirus RGDAd-UPII-TK combined with GCV could suppress bladder cancer in implanted tumor models.

## DISCUSSION

At present, gene therapy-based precision medicine is regarded as a promising procedure for cancer treatment. Since mammalian cell membranes do not allow for active internalization of naked macromolecules, such as nucleic acids and proteins, several classes of viruses, including retroviruses, lentiviruses, herpes simplex viruses, and adenoviruses, have been extensively used as gene delivery vectors [10]. Notably, adenoviral vectors have been showing superior attributes in extensive preclinical and clinical trials with improved specificity and potency [22].

In this study, we generated a novel bladder cancer specific adenovirus by instituting certain genetic modifications. Bladder cancer cells with a decreased expression of CAR are resistant to the commonly used Ad5 infection, and therefore to adenovirus-mediated gene therapy [23]. So we generated a RGD motif in the HI loop of the adenoviral fiber to overcome the CAR deficiency in bladder cancer and optimize the efficiency of therapeutic gene transfer. We also utilized a suicide gene called HSV-TK, the expression of which was restricted to bladder cancer cells under the control of the bladder-specific UPII promoter. The HSV-TK displayed a 1000-fold higher affinity for its substrate GCV than the mammalian TK, and this targeting design could reduce the unwanted HSV-TK activity in other non-bladder cancer tissues, which limits systemic toxicity. We utilized HSV-TK in combination withGCV as a therapeutic strategy but no cytotoxic agent was used alone for the safety of patients. The recombinant adenovirus cannot eliminate cells when it is administered alone. And “bystander effect” can considerably solve the problem of low transfection efficiency in gene therapy.

Because we have generated recombinant RGDAd-UPII-TK, further experiments were performed to examine its therapeutic efficacy for bladder cancer. We demonstrated that the bladder cancer specific infectivity of RGDAd-UPII-TK was efficient, and the suicide HSV-TK gene was highly expressed in T24, BIU-87, and 5637 bladder cancer cell lines. Furthermore, the combination treatment of RGDAd-UPII-TK and GCV could not only eliminate bladder cancer cells *in vitro* but also suppress the implanted bladder cancer tumors and induce more apoptosis *in vivo*. Taken together, our results indicated that RGDAd-UPII-TK combined with GCV was a strategy for bladder cancer therapy.

Although great efforts have been made over the past few years, the adenovirus-induced host immune responses still impact the gene transfer efficacy [24, 25]. Avoiding the systemic administration can minimize the immune system's clearance of adenoviral vectors. Because the bladder is an anatomically accessible organ, the therapeutic adenovirus can come in direct contact with the bladder cancer through intravesicular administration. Furthermore, approximately 80% of newly diagnosed bladder cancer patients have non-muscle-invasive bladder cancer (NMIBC) or carcinoma *in situ* (CIS) [26], so bladder instillation may be clinically effective for RGDAd-UPII-TK and GCV delivery, and the bystander killing effect of phosphorylated GCV can expand the apoptosis of the remaining uninfected bladder cancer cells [18]. Another problem is that the efficacy was poor when the therapeutic adenoviruses were used as standalone therapies, but suitable efficacy was obtained when used in combination with conventional therapies such as chemotherapy and radiotherapy [27]. Meanwhile, over 70% of patients with NMIBC or CIS suffer at least one instance of disease recurrence and progression after successful initial conventional treatment [28]. As a result, future studies will devote the development of combination therapies for bladder cancer.

## MATERIALS AND METHODS

### Cell lines and cell culture

T24, BIU-87, 5637, 293, LNCaP, PC12, A498, HepG2, and BGC823 cell lines were cultured in high glucose DMEM (Gibco) supplemented with 10% fetal bovine serum and 100 U/ml penicillin/streptomycin (Invitrogen) at 37°C in a humidified 5% CO_2_ chamber.

### Modification of the adenoviral fiber region

The cloning procedure for generating the RGD sequence in the HI loop of the adenoviral fiber was performed by overlapping PCR in three steps, and two sets of RGD-related primers were designed: P1, 5′-CTGACTCTTAAGGACTAGTTTCGCGC-3′ and P2, 5′-GATCTCCACGAGTTGTGTCTCCTGT-3′; P3, 5′-AACTCGTGGAGATCCAAGTGCATACTC-3′ and P4, 5′-ACGTAGGATCCATGCATGTTAATTAA-3′. For the first two steps, the adenovirus backbone plasmid pAdEasy-1 was used as a template. The 27241-30218 sequence was amplified using primers P1 and P2, and the 30219-33480 sequence was amplified using P3 and P4. The cycle conditions were both 95°C for 5 min, followed by 30 cycles at 95°C for 30 s, 58°C for 30 s, and 72°C for 4 min, and a final extension at 72°C for 10 min. The cloned fragment 2979 bp upstream of the HI loop coding region was termed RGD1, and the 3283 bp region downstream of it was termed RGD2. The PCR products with the expected size were extracted from 1% agarose gels. Then, RGD1 and RGD2 were mixed 1:1 as templates for PCR using primers P1 and P4. The cycle conditions were 95°C for 5 min, followed by 30 cycles at 95°C for 30 s, 58°C for 30 s, and 72°C for 6 min, and a final extension at 72°C for 15 min. The final PCR products, termed RGD-HI, were digested with the restriction enzymes SPE1 and Pac1 and then inserted into the plasmid pAdEasy-1 at the restriction enzyme cutting sites. Sequence analysis (Beijing Genomics Institution) confirmed that the RGD sequence had been successfully generated.

### Adenoviral vector construction

The UPII promoter was cloned by PCR. T24 genomic DNA was used as a template, and the primers were 5′-ACCGGTCAGGCTTCACCCCAGACC-3′ and 5′-GATGCTGGGCTGGGAGGTGGAATAG-3′. The cycle conditions were 95°C for 5 min, followed by 30 cycles at 95°C for 30 s, 59°C for 30 s, and 72°C for 6 min, and a final extension at 72°C for 15 min. Then, the UPII promoter and HSV-TK gene were inserted into a shuttle vector at multiple cloning sites (MCS) to create pS-UPII-TK. The adenovirus was generated through homologous recombination of the adenoviral backbone plasmid and the shuttle plasmid in the E. coli BJ5183 cells. To amplify and package the recombinant adenovirus, HEK293 cells were transfected with linearized RGDAd-UPII-TK vector by Lipofectamine^™^ 2000 (Invitrogen). After 7 days (in which the cells were passaged three times), the adenovirus particles were harvested and purified by CsCl density gradient centrifugation.

### Reporter gene detecting assay

T24, BIU-87, 5637, 293, LNCaP, PC12, A498, HepG2, and BGC823 cells were plated in a 24-well plate (2 × 10^5^ cells/well) and grown overnight. Then, the cells were infected with a Luciferase recombinant adenovirus RGDAd-UPII-Luc or Ad-UPII-Luc. After 24 h, the cells were treated with 200 μl reporter lysis buffer (Promega), and a freeze-thaw step was performed to ensure complete lysis. Luminescence levels were measured with a luciferase assay kit (Promega) and counted on a Wallac 1450 Microβliquid scintillation counter (Wallac).

### Western blotting

Equal amounts of proteins extracted from cell lysates were separated by sodium dodecyl sulfate-polyacrylamide gel electrophoresis (SDS-PAGE) and then transferred onto the PVDF membrane (Amersham Life Science). The detection of protein was performed using primary antibodies against HSV-TK, β-actin and HRP-conjugated secondary anti-rat antibody (Abcam).

### MTT assay for cell survival analysis

Bladder cancer cells (T24, BIU87, 5637) and other cells (293, PC12, A498, HepG2, BGC823) were plated in 96-well plates (5 × 10^3^ cells/well) and incubated for 24 h at 37°C in a humidified 5% CO_2_ chamber. Then, the cell lines were infected with RGDAd-UPII-TK or RGDAd-UPII-Null. At 24 h post-transfection, the cells were treated or not treated with GCV (0.02 μg/ml). After incubation for 12 h at 37°C, 20 μl of MTT (Sigma) in PBS (5 mg/ml) was added to each well, followed by an additional 4 h of incubation. Subsequently, the supernatant was removed, and 150 μl DMSO (Sigma) was added to each well and mixed thoroughly. The optical density (OD) was measured at 490 nm using a microplate reader. Cell viability was measured as the absorbance of the experimental group compared to the GCV-treated control group.

### BALB/c nude mice and tumor implantations

Six-week-old athymic BALB/c nude male mice weighing approximately 20–24 g were obtained from the Model Animal Research Center of Nanjing University. Mice were quarantined for a minimum of 5 days in the SPF Grade Animal House under 12 h light/dark cycles at 24°C. Guidelines were followed in handling the animals. Tumors were established by subcutaneous (s.c.) injection with T24 cells (1 × 10^6^/100 μl). T24 cells were gently resuspended into 100 μl PBS (pH 7.4; BioSource), mixed 1:1 with Matrigel (BD Biosciences), and injected into the right flank of the mice. Once tumors reached a size of 100–150 mm^3^, the mice were randomized into the control and treatment groups. Adenoviruses were injected into the tumors 3 times (30 MOI at a time) on days 1, 3 and 5. At the same time, GCV was intraperitoneally injected into the mice every day (60 mg/kg/d) for a week. Tumor volumes, based on caliper measurements, were calculated 2 weeks after the injection.

### Histological analysis of tumor tissues

Paraformaldehyde solution-fixed bladder tumor tissues were embedded in paraffin, and sliced into 5-μm sections. The sections were stained with standard hematoxylin and eosin (HE). Images were viewed in a 20× field using a Nikon microscope and were acquired using Image-Pro Plus version 6.2 software (Media Cybernetics).

### Immunohistochemical analysis of apoptosis

Apoptosis of the treated tumor tissues was detected with TUNEL staining. The tissues were fixed in Methacarn solution, embedded in paraffin and sliced into 5-μm sections. Subsequently, the sections were deparaffinized, rehydrated, and subjected to TUNEL staining for 2 h as described in our previous study [11]. The apoptotic rate was calculated as the number of TUNEL-positive cells per field.

### Statistical analysis

All data are expressed as the mean ± SD and were analyzed with GraphPad Prism 5.0 software. The comparisons between the experimental groups and the corresponding control groups were assessed with variance test. *P* < 0.05 was considered to be significant.
